# Supplementary material for: Enhancing our understanding of short-term rental activity: A daily scrape-based approach for Airbnb listings
Source: PLoS One. 2024 Feb 7;19(2):e0298131. doi: 10.1371/journal.pone.0298131 (PMC10849255; doi:10.1371/journal.pone.0298131)
Supplement: S1 Appendix — (DOCX) [file pone.0298131.s001.docx]

## S1. Key timelines and details on retrieving cancellations on ECPs

### S1.1. Scotland lockdown phases

In Scotland, the first national lockdown started on 23^rd^ March 2020 during which people had restricted movements, non-essential shops as well as hospitality and leisure services closed. Tourism activities ceased as a result. The measure was gradually eased from the end of May. From 15^th^ July, non-essential shops were allowed to open again. By the end of July, deaths with confirmed COVID had dropped to zero and remained at or close to that level until September of that year. The first wave of COVID-19 had passed.

### S.1.2. Retrieving scrapes for ECPs

When the first policy was put forward on 16^th^ March 2020. The policy covered the bookings made between 14^th^ March and 14^th^ April. For the listings with the calendars since 16^th^ March, we retrieve the calendar updates that fell within this booking period. We count the number of days being changed from unavailable to available, as days being cancelled, daily until 30^th^ March when the second policy was put forward.

The second policy allowed guests to cancel new bookings, between 15^th^ April and 1^st^ June. Guests not only started to cancel their bookings made for these newly added dates. At the same time, they also placed their cancellations covered by the 1^st^ policy. As a result, for the scraping date from 30^th^ March, on one hand, we continuously retrieve calendar dates covered by Policy 1, that is until 14^th^ April, and count cancellations. On the other hand, we also retrieve cancellations between 14^th^ April and 1^st^ June. Cancellations made towards these dates reflect the impact of Policy 2.

On 1^st^ May, Policy 3 was announced when bookings for 1^st^ June until 15^th^ June were eligible for cancellation. Similar to Policy 2, guests started a new round of cancellations for the two newly identified weeks, meanwhile, guests still had the chance to cancel those days covered by Policy 2. As we go through the scraped calendars, from 1^st^ May, we retrieve cancellations between 1^st^ May and 1^st^ June for Policy 2 while 1^st^ June to 15^th^ June for Policy 3.

From the 1st of June, Policy 4 covers more days to the 15^th^ of July. To identify cancellations which are covered by Policy 3, we use scrapes dated from 1^st^ June, retrieving the first part of calendar cancellations until the 15^th^ of June. We also retrieve a second part from 15^th^ June to 15^th^ July accounting for the impact of Policy 4.

Finally, for the scrapes made after 15^th^ June, we retrieve an additional period, between 15^th^ July and 30^th^ July, to identify changes as a result of Policy 5 as well as track cancellations made between 15^th^ June and 15^th^ July for the coverage of Policy 4.

## S2. Tracking noise may affect estimations

Market activities have two sides, as shown in Figure S2.1. Our method collected the following information: (a) the total number of days changed to available shown in the upper blue pane, (b) the total number of days being changed to unavailable shown in the bottom green pane. The total number of calendars observed every day is denoted in red. There are discontinuities in scraping, particularly in the early days of the pandemic, which means that the number of listings with consecutive calendars can be lower than reality.


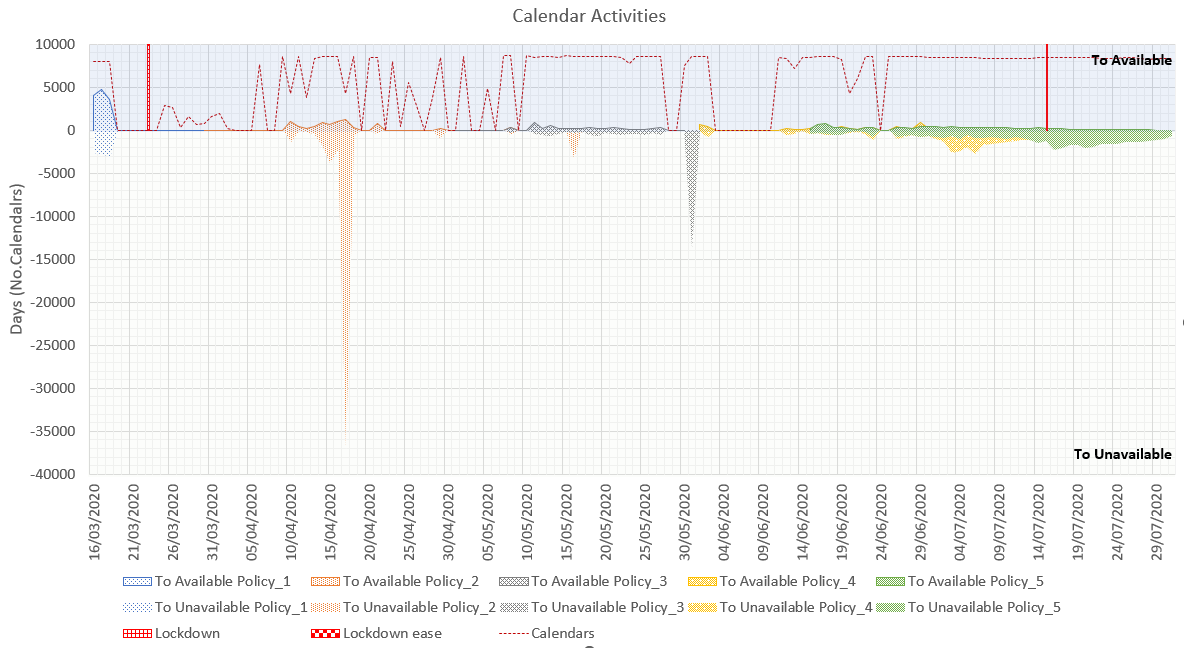


Fig S2.1. Summary of overall calendar activities in the study period.

While not the primary focus of this paper, Figure S2.1 sheds light on the opposite side of the market, where specific calendar days are deactivated for booking. The spikes in the data indicate that there may be corporate actions taken in response to market disruptions, resulting in the deactivation of certain days for new bookings.
