# Supplementary material for: Enhancing our understanding of short-term rental activity: A daily scrape-based approach for Airbnb listings
Source: PLoS One. 2024 Feb 7;19(2):e0298131. doi: 10.1371/journal.pone.0298131 (PMC10849255; doi:10.1371/journal.pone.0298131)
Supplement: Attachment — Submitted filename: Manuscript Number PONE-D-23-19176 - Google Docs.pdf [file pone.0298131.s003.pdf]

Review
Manuscript Number: PONE-D-23-19176
Title: "Enhancing our Understanding of Short-term Rental Activity: A Daily Scrape-based Approach for Airbnb Listings"

The paper titled "Enhancing our Understanding of Short-term Rental Activity: A Daily Scrape-based Approach for Airbnb Listings" presents an interesting and valuable approach for studying short-term rental (STR) activity, specifically focusing on Airbnb listings. The authors address the challenge of data scarcity and quality in studying the impact of the online short-term rental market on housing supply and policy interventions. The paper proposes a methodology involving daily web scraping of Airbnb calendars to derive fine-grained data, aiming to provide better insights into booking activities, occupancy rates, and market trends. The methodology is demonstrated through its application to the Airbnb market in Edinburgh during the COVID-19 pandemic.

Strengths:

Original Approach: The paper's approach of daily web scraping to collect detailed data from Airbnb calendars is a novel and innovative way to address the limitations of existing data sources. This approach can potentially offer a more accurate and temporally detailed understanding of STR activity, which is crucial for policy evaluation and market analysis.

Relevance: The topic of short-term rentals and their impact on housing supply and urban policies is highly relevant, especially in the context of platforms like Airbnb. The paper's focus on providing data-driven insights to aid policy interventions is important for informed decision-making.

Data Transparency: The authors emphasize the importance of data transparency and make their codebase openly available on GitHub, enhancing the reproducibility and credibility of their research. This practice promotes collaboration and peer review within the research community.

Real-world Application: The application of the proposed methodology to analyze the effects of "Extenuating Circumstances Policies" (ECPs) during the COVID-19 pandemic provides a practical demonstration of the approach's value. The case study in Edinburgh adds real-world context to the research.

Weaknesses:
-Data Quality and Reliability: The paper emphasizes data quality but does not thoroughly address issues related to data breaks, missing data, and the potential impact of data quality on the analysis. Discussing potential sources of bias and limitations due to data quality would enhance the paper's rigor.
Please discuss about:
>>Describe the strategies implemented to address or mitigate issues related to data quality, including handling data breaks and missing data. This could involve discussing imputation methods or data validation procedures.
>>Present a clear analysis of the potential impact of data quality issues on the results. This could involve sensitivity analyses that assess how variations in data quality might affect key findings.

-Platform and Contextual Changes: The study focuses on a specific time period (COVID-19 pandemic) and a specific location (Edinburgh). The discussion should include considerations about the generalizability of findings to other time periods, locations, and different regulatory contexts.
Please discuss about:

>>Address the potential influence of platform changes (such as modifications to Airbnb's calendar format) on the data collection and analysis process. Explain how the proposed methodology adapts to such changes and maintains its accuracy.
>>Discuss the transferability of the approach to different locations and time periods, along with potential challenges and adjustments required to apply the methodology in diverse contexts.

Conclusion:
The paper presents a valuable approach to address the limitations of existing data sources in studying short-term rental activity, particularly in the context of Airbnb. The methodology's potential benefits for policy evaluation and market analysis are clear. However, to strengthen the paper, it is essential to address the weaknesses mentioned above. More in-depth discussions of the methodology's limitations, data quality issues, and the broader applicability of findings would provide a more comprehensive understanding of the approach's strengths and limitations. Additionally, further explanation of assumptions, potential biases, and strategies to handle missing data would enhance the paper's credibility and usefulness to both researchers and policymakers.
